# PSNet: A Deep Learning Model-Based Single-Shot Digital Phase-Shifting Algorithm

**DOI:** 10.3390/s23198305

**Published:** 2023-10-08

**Authors:** Zhaoshuai Qi, Xiaojun Liu, Jingqi Pang, Yifeng Hao, Rui Hu, Yanning Zhang

**Affiliations:** 1 College of Computer Science, Northwestern Polytechnical University, Xi’an 710072, China; xj_liu@mail.nwpu.edu.cn (X.L.); jingqip@mail.nwpu.edu.cn (J.P.); hyifeng@mail.nwpu.edu.cn (Y.H.); 18292376709@163.com (R.H.); ynzhang@nwpu.edu.cn (Y.Z.); 2 National Engineering Laboratory for Integrated Aero-Space-Ground-Ocean Big Data Application Technology, Xi’an 710072, China

**Keywords:** fringe projection, relative phase retrieval, phase shifting, deep learning, 3D reconstruction

## Abstract

In contrast to traditional phase-shifting (PS) algorithms, which rely on capturing multiple fringe patterns with different phase shifts, digital PS algorithms provide a competitive alternative to relative phase retrieval, which achieves improved efficiency since only one pattern is required for multiple PS pattern generation. Recent deep learning-based algorithms further enhance the retrieved phase quality of complex surfaces with discontinuity, achieving state-of-the-art performance. However, since much attention has been paid to understanding image intensity mapping, such as supervision via fringe intensity loss, global temporal dependency between patterns is often ignored, which leaves room for further improvement. In this paper, we propose a deep learning model-based digital PS algorithm, termed PSNet. A loss combining both local and global temporal information among the generated fringe patterns has been constructed, which forces the model to learn inter-frame dependency between adjacent patterns, and hence leads to the improved accuracy of PS pattern generation and the associated phase retrieval. Both simulation and real-world experimental results have demonstrated the efficacy and improvement of the proposed algorithm against the state of the art.

## 1. Introduction

In optical interferometry [1] as well as in 3D reconstruction using fringe projection profilometry (FPP) [2,3], phase retrieval is a common but fundamental step that often consists of two stages: wrapped or relative phase retrieval and phase unwrapping. While extensive efforts have been made to reduce phase errors induced via gamma nonlinear error elimination [4], color cross-talk [5], highlights [6], interreflection [7] and motion [8], which leads to improved phase accuracy, the efficiency is still unsatisfactory for real-time applications due to the projection of multiple fringe patterns with different frequencies and phase shifts [9]. For instance, during relative phase retrieval with a standard N-step phase-shifting (PS) algorithm, at least N ≥ 3 fringe patterns with different phase shifts are needed, which limits the algorithm to a relatively narrow range of applications. In particular, capturing multiple patterns in FPP makes relative phase retrieval more prone to ambient disturbances, as well as limiting it to static scenarios. Additionally, the two-stage process (relative phase retrieval followed by phase unwrapping) for absolute phase retrieval is too cumbersome.

To reduce the number of fringe patterns, single-fringe-pattern-based phase retrieval methods have been proposed. For instance, composite fringe projection methods such as those proposed by [10,11] try to embed multiple fringe patterns with different PS or frequencies, or to embed spatial coded patterns in a single pattern. With some demodulation methods, these embedded patterns can be decomposed from the captured composite pattern, and are then used for relative phase retrieval and unwrapping. However, the embedding of additional patterns may reduce the spatial resolution of the phase or reliability during decomposition. Therefore, more advanced algorithms have been developed to extract absolute phases directly from a single-shot, single-frequency fringe pattern. Typical works include the untrained deep learning-based method [12] and wavelet-based deep learning method [13]. The former achieves absolute phase retrieval with two networks, where the first one refines the relative phases and produces a coarse fringe order for unwrapping and the second one unwraps the relative phases with the fringe order and then refines them. While this achieves absolute phase retrieval with only a single-shot pattern, at least two cameras are required, which increases the system’s complexity and cost. The wavelet-based method combines both wavelet and deep learning techniques, where the wavelet provides a preprocessing tool to enhance speed while deep learning tries to directly estimate the depth of a scene. The speed of the process has been increased significantly via this method. However, considering the insufficient information in only one fringe pattern for absolute phase retrieval, the performances of these single-shot methods are still unsatisfactory for practical applications.

In contrast to absolute phase retrieval, relative phase retrieval is much easier and thus single-shot methods have been the focus in related fields. The traditional PS method has already achieved satisfying accuracy in relative phase retrieval, which is less efficient due to the projection of patterns with different phase shifts. To reduce the number of PS steps, a two-step PS algorithm has been presented, where only two patterns are required. To further reduce the patterns, digital PS algorithms with only a single fringe pattern have been proposed. In a typical work, a digital four-step PS algorithm was developed based on Riesz transform (RT) [14]. Given only one fringe pattern, three π/2 phase-shifted patterns can be generated using the RT algorithm. With the resulting four-step PS patterns, phases can be retrieved pixel-wisely using conventional PS algorithms. While the RT algorithm performs well for patterns with a high signal-to-noise ratio (SNR), the performance decreases dramatically in the case of degenerated patterns with variations in surface curvature and reflectance. Additionally, the number of generated PS steps is limited to four due to the π/2 phase shift, which makes the algorithm more sensitive to noise. Finally, a single-shot N-step PS method [15] has been proposed. With an algebraic addition and subtraction process, arbitrary-step PS patterns can be generated. However, since this method relies on Fourier transform, it may perform poorly on the sharp edges of an object. 

To enhance the phase quality of sharp edges or in situations of discontinuity, deep learning-based relative phase retrieval methods have been proposed. Assuming a mapping between the phase and fringe pattern, a two-stage algorithm [16,17] was used to try to solve this as a typical regression task. More specifically, it was used to try to regress an intensity map from a single fringe pattern in the first stage; guided by this, the wrapped phase was then estimated via regressing the numerator and denominator from the same pattern in the second stage. To achieve further improvement, a new loss in the predicted modulation of the fringe was introduced in [18]. Building upon these methods, the influence of the basic U-Net network structure and hyper-parameters was investigated [19], and inspired the establishment of better structures and parameter settings for improved accuracy. These methods have also been extended to scenarios containing multiple stages [20] or patterns [21], demonstrating promising performance. These kinds of methods are among the earlier and representative works using deep learning in fringe analysis. However, since mapping is a significant ill-posed problem, directly learning mapping from the fringe to the phase (or equivalently from the numerator to denominator) without additional constraints is still challenging for a deep learning model.

To simulate PS and achieve pixel-wise relative phase retrieval, researchers proposed a one-stage algorithm called FPTNet [22]. In contrast to the aforementioned two-stage approaches, FPTNet treats relative phase retrieval as an image generation task. It tries to generate N-step PS patterns using only a single pattern. This novel technique used in [22] enables us to naturally leverage the additional and abundant constraints in the intensity of PS fringe patterns. Consequently, this algorithm has demonstrated improved performance in relative phase retrieval. However, it is worth noting that these methods often focus on intensity loss during model training, which captures only local temporal information for fringe generation. Less attention, however, has been paid to the temporal dependency between patterns, which models the global temporal information. Therefore, there remains room for further improvement.

In this paper, we followed the ideas of “image generation” and proposed a deep learning model-based digital PS algorithm, termed PSNet, which can accurately predict other PS patterns from a single pattern. Our method incorporates both local and global temporal information during the training process, thus helping to learn inter-frame dependence between adjacent patterns. Consequently, it enables more accurate digital N-step PS generation and hence enhances relative phase retrieval. The main contributions can be summarized as follows.

(1) The proposed PSNet allows for the generation of N-step PS patterns using only one pattern. Additionally, the relative phases can be retrieved in a pixel-by-pixel fashion with a typical PS algorithm, which thus performs more robustly for regions with phase discontinuity.
(2) Unlike previous works that rely only on image intensity loss (typically regraded as local temporal information), our method incorporates both local and global temporal information in the predicted fringe intensity, which significantly improves the accuracy of relative phase retrieval.
(3) Since a single fringe pattern is sufficient for relative phase retrieval, the efficiency of the PS algorithm can be improved, which will benefit its real-time application.

## 2. Fundamental Principle of the Proposed Algorithm

### 2.1. Phase Shifting Technique

A typical captured fringe pattern with a phase shift is expressed as

(1) $$I_{i}(x,y)=a(x,y)+b(x,y)\cos\left[\varphi (x,y)+\delta (i-1)\right]$$

where *a*, *b* and *φ* are the average intensity, modulation and wrapped phase of the captured fringe pattern, respectively. (*x*, *y*) denotes the camera image coordinate. *δ* is the phase shift, and *i* = 1, …, *N* indicates the pattern with an *i*-th phase shift. *N* is the total number of phase shifts.

With the conventional PS algorithm, the wrapped phase, *φ*, can be retrieved using

(2) $$\varphi (x,y)=-\arctan\frac{S}{C}=-\arctan\frac{\sum_{i=1}^{N}I_{i}\sin(\delta i)}{\sum_{i=1}^{N}I_{i}\cos(\delta i)}$$

where *S* and *C* are the sine and cosine summations, respectively.

### 2.2. Architecture of the Proposed PSNet

**Figure 1:**
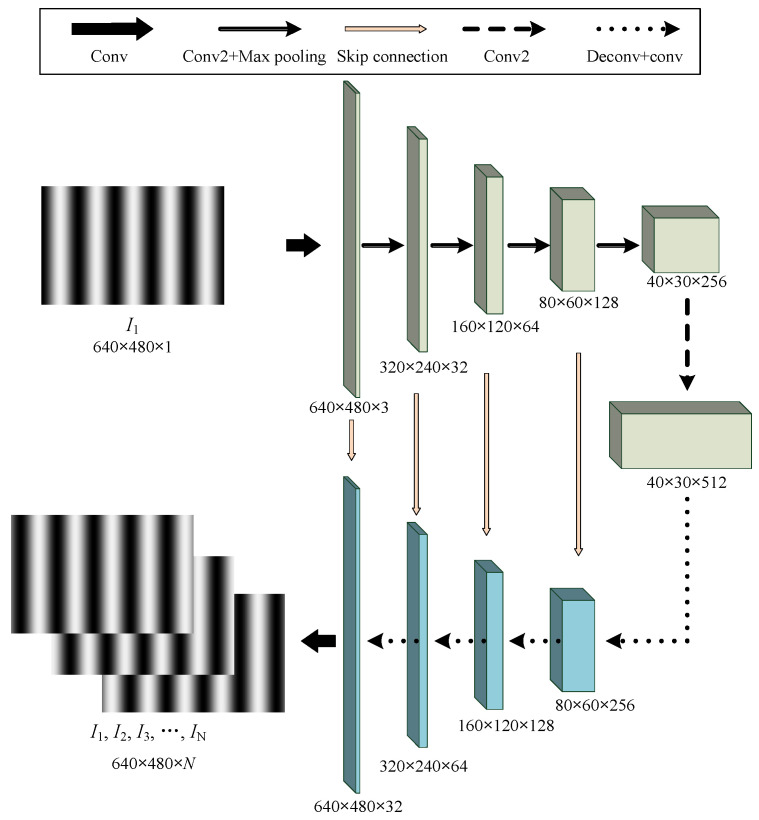
Architecture of PSNet.

According to Equation (2), at least N ≥ 3 patterns are required for relative phase retrieval, which means it is time-consuming to capture them. To this end, we propose a deep learning model, called PSNet, to predict patterns *I*_i_ *i* = 2, …, *N* with different PSs from a single fringe pattern, *i.g. I*_i_, *i* = 1. The model is constructed with a typical Unet [23] as the backbone. The detailed architecture of PSNet is shown in Figure 1.

As shown in Figure 1, the input of PSNet is a single fringe pattern with 640 × 480 pixels (denoted as *I*_1_), and the output is the sequence of predicted *N* PS patterns, i.e., *I*_i_, *i* = 1, 2, 3, …, N. please note that *I*_1_ is also included in the output, but it can be optionally removed. PSNet has an approximatively symmetric architecture, as shown in Figure 1. Near the input end, there is one “Conv” layer, four “Conv2 + Max pooling” layers and one “Conv2” layer, which forms the “down sampling” process and produces a 40 × 30 × 512 feature map. Conv represents a typical convolutional layer. Conv2 means performing convolution operation twice sequentially on its input, and“Conv2 + Max pooling” indicates a layer combining Conv2 and max pooling operations. In contrast to the down-sampling process, the up-sampling process includes four Deconv + conv layers and one Conv layer. Deconv + conv layer firstly performs deconvolution operation on the input, and its output is then concentrated with the Skip connection’s output before being processed by a convolutional layer. All convolution kernels have a size of 3 by 3.

### 2.3. Learning Temporal Dependency among the Predicted Sequence

We observe that a sine/cosine wave can be fitted to the intensity of each pixel with different PSs. This implies a strong temporal dependency between the predicted PS patterns, which provides an additional constraint to digital PS learning. Therefore, we have designed a loss function to enforce PSNet to learn such a temporal dependency, which is expressed as

(3) $$L_{\mathrm{temp}}={\left\|S-S^{*}\right\|}^{2}+{\left\|C-C^{*}\right\|}^{2}$$

where S* and C* are the ground truth and *S* and *C* are predicted ones in Equation (2).

Since *S* and *C* are the summation of intensity with different PSs, as shown in Equation (2), encoding temporal dependency among the sequence, the designed loss *L*_temp_ can succesfully guide PSNet to learn the temporal information during training.

Other than the mentioned loss function, we also incorporate intensity loss, *L*_intensity_, into the overall loss, *L*, as follows:

(4) $$L=L_{\mathrm{intensity}}+\lambda L_{\mathrm{temp}}$$

where *λ* is a weight factor. *L*_intensity_ is expressed as

(5) $$L_{\mathrm{intensity}}=\sum_{i=1}^{N}{\left\|I_{i}-I_{i}{}^{*}\right\|}^{2}$$

These two types of information, intensity and temporal dependency, contribute to a more accurate prediction of PS patterns.

### 2.4. Phase Unwrapping for Absolute Phase Retrieval

Since only wrapped phase of the fringe can be retrieved from the digital phase-shifting patterns generated from our method, an additional unwrapping algorithm is needed to unwrap them to absolute phases, which can then be used for 3D reconstruction. A typical unwrapping approach based on a multi-frequency fringe pattern is explanation as follows. 

Given the wrapped phases, *φ*^w^_k_, of different-frequency fringes, where *k* = 1, 2, …, *M*, phase unwrapping can be achieved sequentially as follows:

(6) $$\varphi_{k}^{\mathrm{uw}}(x,y)=\varphi^{\mathrm{uw}}(x,y)+\mathrm{Round}\left(\frac{\varphi_{k}^{\mathrm{uw}}\frac{f_{k}}{f_{k-1}}-\varphi_{k}^{w}}{2\pi }\right)2\pi$$

where, *φ*^uw^_k_ is the unwrapped phase of the fringe with frequency, *f*_k_. *f*_M_ > *f*_M−1_ > *f*_M−2…_ > *f*_k_ > *f*_k−1…_ > *f*_2_ > *f*_1_ (where *k* = 1, 2, …, and *M*) are the frequencies of the multi-frequency fringes, and *M* is a positive integer representing the total number of frequencies. There is only one fringe in the pattern with frequency, *f*_1_, and there is no phase wrapping; thus, *φ*^uw^_1_ = *φ*^w^_1_. Round(x) is the round function.

By utilizing at least two frequencies, we can obtain the absolute phase from the wrapped phases. To evaluate the performance of our method, both the wrapped phases and the absolute phases after unwrapping are considered. The retrieved wrapped phases from our method are further unwrapped using the mentioned unwrapping algorithm, resulting in the absolute phases. More details regarding the fringe frequencies can be found in the experiment section (Section 4).

### 2.5. Dataset and Training

**Figure 2:**
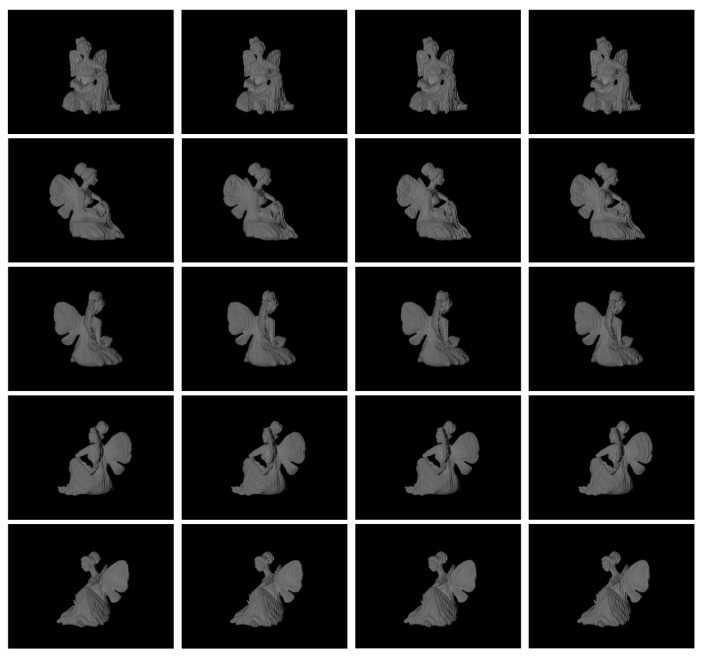
Example of an object in the dataset, where each column represents patterns with different phase shifts, i.e., *I*_i_, *i* = 1, 3, 5, 7, and each row represents the object in different poses.

**Figure 3:**
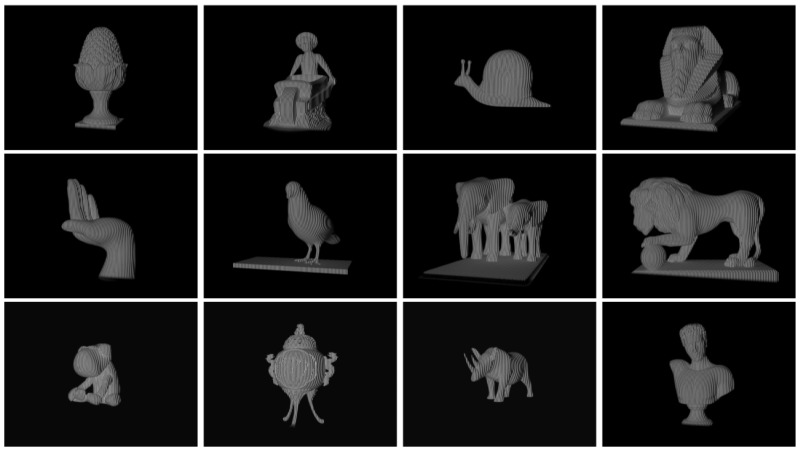
Samples in the dataset.

To the train the proposed model, we introduced a simulated PS pattern dataset consisting of approximately 180 objects, which cover diverse shapes, reflectance and poses. In our simulation environment, we implemented a FPP system of one 640 × 480 pixel camera and a 1280 × 800 pixel projector. With this FPP, we captured a set of eight-step PS patterns for each object in a particular pose. This set of patterns was considered one training sample. We collected five samples for each object in different poses, as shown in Figure 2. Consequently, our dataset consists of 900 samples, some of which are shown in Figure 3.

The training of the our model was implemented in Pytorch [24]. After approximately seven epochs, the performance of training tended to stop improving, and accordingly, we stopped training after 20 epochs. During training, data augmentation was applied to overcome sensitiveness to varying surface curvatures, reflectance, and lighting conditions in the real scenario. This further enhanced the generalization of PSNet. Specifically, each sample was augmented randomly in accordance with the following equation:

(7) $$I_{augmented}=kI_{i}+I_{\mathrm{offset}}$$

where *k* and *I*_offset_ are the scale factor and intensity offset, respectively, which are selected randomly from ranges [0.5, 1.2] and [−0.05, 0.05], respectively.

The simulation environment was implemented using the 3Ds Max 2018 platform [25]. As one of the mainstream simulation platforms, 3Ds Max accurately simulates the imaging processes of physical cameras and those of image projection. Furthermore, like most of similar works [26,27], constructing a simulated dataset is “cheaper” than capturing images in real world is. Before training, the dataset was spilt into training, validation and testing sets in a ratio of 8:1:1. This resulted in approximately 720 samples for training, and 90 samples each for validation and testing. The training and testing of the proposed model were performed on a computer with Intel Core i9-10900X@3.70 GHz, a 32 GB RAM and a Nvidia GeForce RTX3080Ti card.

## 3. Results

We evaluated the performance of our algorithm on both simulation and real-world data. For comparison, we also implemented the two-stage algorithm [15] and FPTNet [22] as the baselines. The captured fringe patterns and their retrieved phases and reconstruction results were regarded as the ground truth (GT). Compared to the baselines, our algorithm demonstrated comparable and even superior performance in both fringe generation and phase retrieval. The results are provided as follows.

### 3.1. Evaluation on Simulation Data

**Figure 4:**
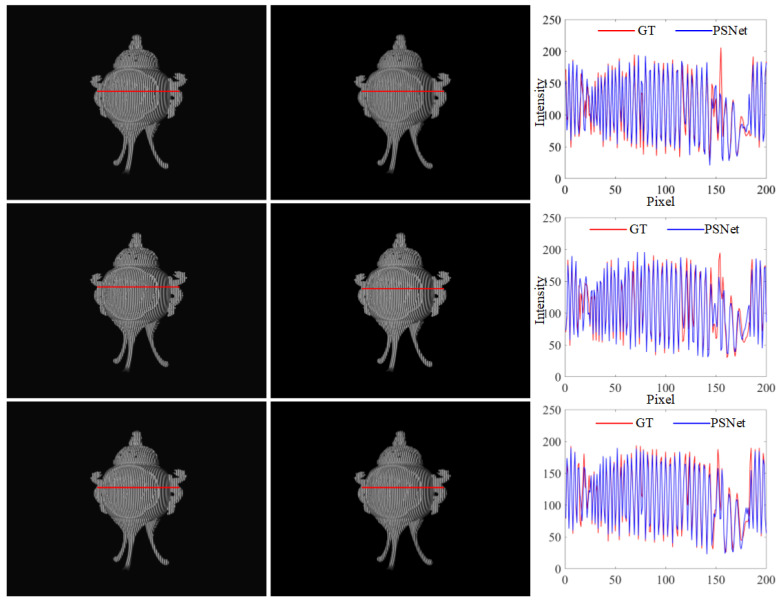
PS patterns of a tripod: (from left to right) results of the ground truth and PSNet, and their cross-sections whose positions are marked by red lines in the first two columns; (from top to bottom) fringe patterns with PS π/2, π and 3π/2.

Simulation was performed based on the same FPP system used during the generation of the training data. The FPP system consisted of a camera with 640 × 480 pixels and a projector with 1280 × 800 pixels. The fringe frequency and number of phase shift steps of projected fringe patterns were 1/10 and four steps, respectively. The captured fringe patterns and predicted ones are shown in Figure 4. It is evident that the fringe patterns predicted via PSNet are visually identical to the GT. Furthermore, from a closer look at the cross-sections, we can also observe that the intensity distribution of the predicted fringe is very close to the GT, except for a few regions with random errors, as shown in Figure 4. The results above suggest the promising performance of the proposed PSNet in the accurate prediction of PS patterns.

**Figure 5:**
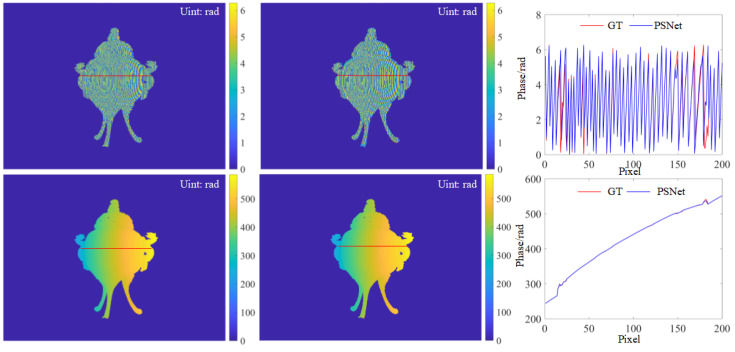
Retrieved phases: (from left to right) the GT, and result of PSNet and cross-sections, whose positions are marked by red lines in the former two columns; (from top and bottom) wrapped and unwrapped phases.

For further validation, an additional comparison of the retrieved phase was performed. As shown in Figure 5, the wrapped and unwrapped phases retrieved using the predicted four-step PS patterns are also very close to the GT. Similarly, only minor differences between the results of the proposed PSNet and GT can be observed from the comparison of cross-sections in Figure 5. Even in regions with noticeable phase jumps in the cross-section of the unwrapped phase, the proposed algorithm also achieved comparable performance, providing evidence of the effectiveness of the proposed PSNet. Please note that the unwrapped phase was retrieved using the multi-frequency fringe phase unwrapping (MFPU) algorithm [28], where the projected fringe frequencies for unwrapping were 1/80 and 1/1280, respectively.

**Figure 6:**
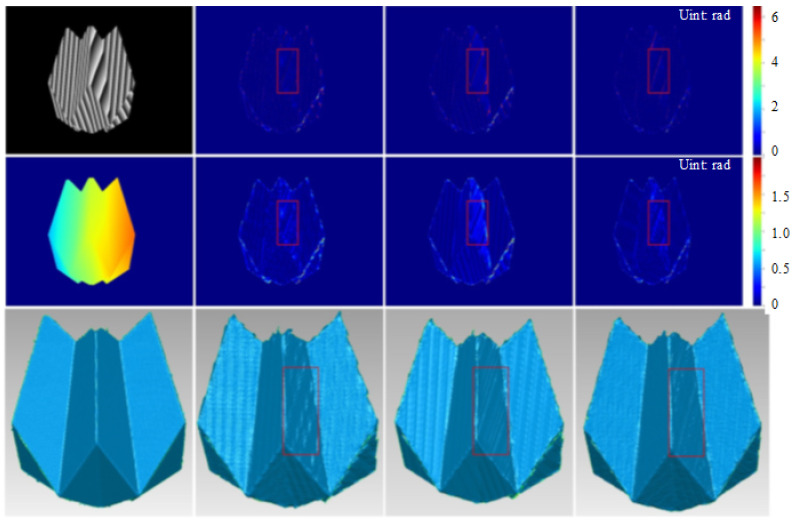
Comparison with state-of-the-art algorithms: (from left to right) results of the ground truth, two-stage algorithm, FPTNet and ours; (from top to bottom) wrapped phase, unwrapped phase and reconstruction results. Regions with obvious errors are marked with a red box.

We also compared our results against the baselines. As shown in Figure 6, the retrieved phases and reconstruction results using our algorithm remain highly consistent with those obtained via the two-stage algorithm and FPTNet. Additionally, obvious phase and reconstruction errors can be observed in regions with a relatively large slope in the results of the baselines, as shown in regions in a red box in Figure 6. In contrast, the errors in our results are considerably smaller, demonstrating the higher accuracy of our algorithm compared to that of the baselines.

### 3.2. Evaluation on Real Data

To validate the performance of our method in real-world applications, we conducted extensive experiments in various scenarios and obtained a real-world dataset for validation. We implemented a FPP system of a 3384 × 2704-pixel camera and 1280 × 800-pixel projector to capture samples for the dataset. The frequency and number of phase shift steps for the fringe patterns used were 1/10 and eight, respectively. The captured patterns with 0 PS were fed into our PSNet model, while the patterns with different PSs (i.e., *i*π/8, *i* = 1, 2, …, 7) were treated as the ground truth. The phases retrieved using the phase-shifting algorithm with the captured fringe patterns were also considered the ground truth. The captured patterns were resized from 3384 × 2704 to 640 × 480 to fit the input format of PSNet. Similarly, additional patterns were projected for phase unwrapping, where fringe frequencies were 1/80 and 1/1280, respectively. The dataset consisted of fringe patterns captured on diverse objects, where the results of the three most representative objects such as boxes (with simple planes), walnuts, and a statue with complex freeform surfaces are presented. Please note that the real-world dataset was only used for evaluation and was not used during the training of the PSNet.

**Figure 7:**
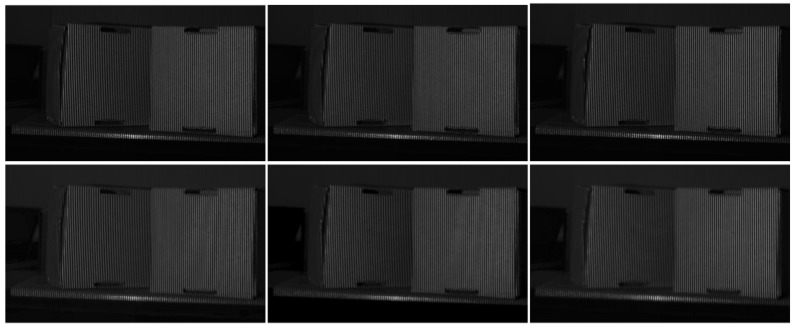
PS patterns of boxes: (from left to right) patterns with phase shift π/2, π and 3π/2, and (from top to bottom) the results of the GT and ours.

**Figure 8:**
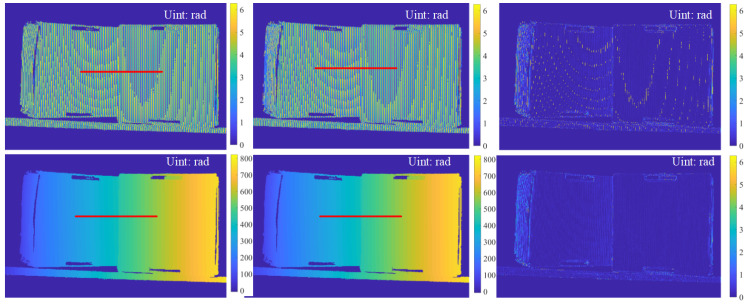
Phases and errors: from left to right, each column represents the GT, the result of the PSNet and the phase error maps; the top and bottom rows represent wrapped and unwrapped phases, respectively.

**Figure 9:**
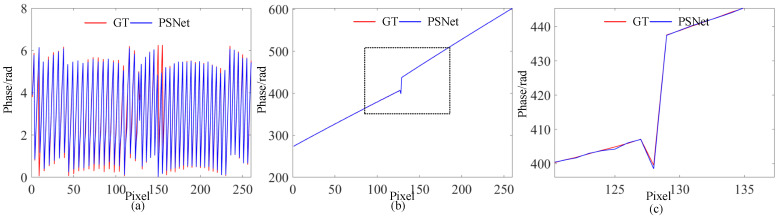
Cross-sections (**a**) and (**b**) are of wrapped and unwrapped phases marked with red lines in Figure 8, and (**c**) is the zoomed-in version of the curve boxed in a dashed black line in (**b**).

The results of two boxes are shown in Figure 7 and Figure 8. Similarly, there are no noticeable differences between the predicted patterns and the GT, as shown in Figure 7. Furthermore, comparable phase retrieval results were achieved using the predicted patterns, as shown in Figure 8, with only small phase errors in the phase error maps. To demonstrate the performance on discontinuous surfaces with large phase jumps, we compared cross-sections of the phases marked with red lines in Figure 8. As shown in Figure 9, PSNet demonstrated comparable performance for both wrapped and unwrapped phases in comparison to the GT. In particular, around the discontinuity between the two boxes, there is an obvious phase jump, where the proposed PSNet performed as well as the GT did with only minor deviations. Th results above validate the effectiveness and generalizability of our proposed algorithm in real-world complex scenarios with significant discontinuities.

**Figure 10:**
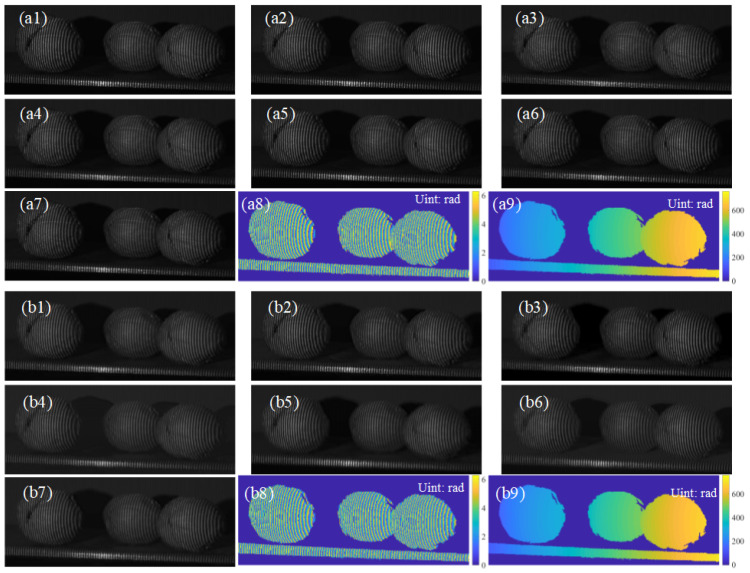
Eight-step PS patterns and retrieved phases; (**a1**–**a7**) are GT patterns with phase shifts π/4, π/2, 3π/4, π, 5π/4, 3π/2 and 7π/4, respectively, and (**b1**–**b7**) are predicted ones; (**a8**,**a9**) are wrapped and unwrapped phases, and (**b8**,**b9**) are those of the PSNet.

We also demonstrated the capability of our algorithm in generating arbitrary-step PS patterns. As shown in Figure 10, it is obvious that PSNet-predicted PS patterns are visually identical to the GT, and additionally, achieved accurate relative phase retrieval compared to the GT results.

**Figure 11:**
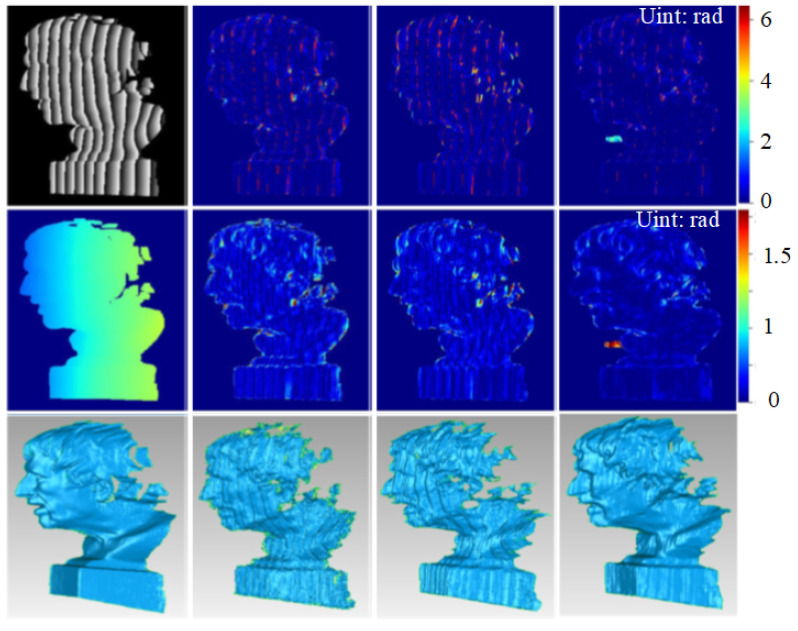
Comparison with the baseline (from left to right). Results of the ground truth, two-stage algorithm, FPTNet and ours; (from top to bottom) wrapped phase, unwrapped phase and reconstruction results.

Similarly, we further compared our algorithm with the baselines, and the results are shown in Figure 11. As expected, our algorithm still performed well for real-world complex data. Compared with the baselines, our algorithm significantly improved phase retrieval accuracy and reconstruction quality, demonstrating superior performance.

To further evaluate the performance quantitatively, we also provided comparison results with respect to the following two aspects: the accuracy of fringe pattern generation and accuracy of phase retrieval.

To assess the accuracy of fringe pattern generation, we used both the peak signal-to-noise ratio (PSNR) and structural similarity index (SSIM) [29], the two mostly used image quality metrics, to comprehensively and quantitatively measure the image similarity between the predicted pattern and the ground truth. Higher values of PSNR and SSIM indicate better similarity and hence better performance. As shown in Table 1, both the baseline and our algorithm performed well, with a PSNR and SSIM greater than 40 and 97%, respectively, indicating the quite-good-quality generation of fringe patterns. In addition, in contrast to the baseline, which was trained only on the image intensity loss, our method achieved an improvement in image quality because it learnt both global and local temporal dependency between the generated patterns. This verifies the efficacy of our proposed method.

For the evaluation of phase accuracy, we used the mean absolute error (MAE) to characterize the phase error. The lower MAE indicates higher accuracy. The results are shown in the last column in Table 1. Similarly, both methods achieved high phase retrieval accuracy, with our method achieving improved accuracy compared to that of the baseline. This further validates the effectiveness of introducing global and local temporal dependency supervision.

We further compared the processing time (PT) of the traditional N-step phase-shifting (PS) algorithm, FPTNet and our method. We processed around 30 samples and then calculated the average PT for each method as its result. More specifically, for the traditional PS algorithm, the PT for each sample only covered the time cost for phase retrieval, and the PTs for 30 samples were averaged to produce the final processing time. For the FPTNet and method, the PT for one sample included the time costs for both fringe pattern generation and phase retrieval, and then, similarly, the average PT for 30 samples was used as the result. All these comparisons were conducted using the same hardware.

The results are shown in Table 2. As we can see, while our method costed the most time compared to the other two counterparts, reaching 0.07 s, the PT of our method remained relatively efficient, achieving a processing speed of 14FPS, which is promising for (quasi) real-time applications.

## 4. Discussion

According to the above experimental results, the proposed method has demonstrated promising performance in relative phase retrieval with a single-shot fringe pattern. This implies that the time required for image acquisition can be reduced since only one fringe pattern is needed, demonstrating potential for real-time 3D reconstruction applications. However, the accuracy of relative phase retrieval with our method is still lower compared to that of the traditional PS algorithm. There is still room for accuracy improvement. In addition, with a PSNet trained on a specific dataset with N-step PS patterns, say N = 8, the PSNet can only generate patterns with the same number of PS steps. To generate PS patterns with a different number of steps (e.g., N = 12), the network needs to be fine-tuned or completely re-trained on the N = 12 PS patterns. This process may be cumbersome for practical applications, since the additional retraining may take several hours. Therefore, a more advanced network which is capable of arbitrary-step PS pattern generation after being trained only once on a dataset of specific-step PS patterns needs to be further investigated.

## 5. Conclusions

A deep learning model-based digital phase-shifting algorithm termed PSNet is proposed in this paper. Other than previous works using only intensity information for supervision, which only captures local temporal information, our method attempts to extract both local and global temporal information from the generated PS patterns. The long-term or global temporal dependency between patterns provides an additional constraint for the PSNet to learn the digital generation process of phase shifting, which consequently contributes a performance improvement to both the quality of the generated patterns and the accuracy of retrieved relative phases. Simulation and real-world data both demonstrate the comparable and even superior performance of the proposed algorithm compared to that of the state of the art. The proposed algorithm can be widely used in phase retrieval in FPP, and can significantly reduce the time costs of capturing multiple patterns.

## Figures and Tables

**Table 1:** Quantitative comparison of image similarity and phase accuracy.

Methods	Image Similarity		Phase Accuracy
	PSNR/dB	SSIM/%	MAE/Rad
FPTNet	41.3	97.2	0.217
Ours	43.5	98.2	0.133

**Table 2:** Comparison of processing time cost.

Methods	Traditional PS	FPTNet	Ours
Processing time/s	0.03	0.04	0.07

## Data Availability

The data presented in this study are available on request from the corresponding author.
